# So-Called Ulceration of the Womb

**Published:** 1877-03

**Authors:** Haywood Smith

**Affiliations:** Physician to the Hospital for Women, and to the British Lying-in Hospital


ON SO-CALLED ULCERATION OF THE WOMB.—*By Haywood Smith, M.A., M.D., Oxon, Physician to the Hospital for Women, and to the British Lying-in Hospital.*—It is a pity that, notwithstanding that the College of Physicians have given us a nomenclature of disease fully up to our present knowledge, and compiled with the greatest care, yet that medical men will persist in the use of terms that only mislead in their description of diseases, and whose existence should be delegated to the region of exploded pathology. One of the most common mistakes is that of calling granular inflammation of the lips of tbe uterus "ulceration." The mistake arises from the desire of giving the public a name for a disease that may easily be remembered, regardless of the pathological accuracy of the description; and, I fear, not unfrequently, from the wish, on the part of the practitioner, to enhance the magnitude of his cure by giving the patient an incorrect idea of the serious nature of her malady.

True ulceration of the cervix uteri is a disease of extreme rarity. It may present itself to us as strumous ulcer, or a syphilitic ulcer, which in its primary manifestation on the lips of the womb is also very rare, or as a rodent malignant ulcer; whereas the condition commonly called "ulceration of the womb" is really "granular inflammation of the cervix," and is one of the most common diseases that fall under the observation of gynæcologists in the out-patient room.

It is characterised usually by a patch or ring of red, bleeding granulations on one side of, or surrounding the os uteri, the cervix is swollen and somewhat tender, the os generally more or less patent, and exuding the glairy discharge characteristic of endocervicitis.

To the touch it gives the sensation of a velvety tissue, in most cases easily distingnishable from the normal smooth surface of the labia uteri.

It rarely is found in virgins, but very frequently in the married, and more especially in those who have borne children.

The symptoms are continuous backache (sacral), with a sense of weight and bearing down; dysmenorrhœa by the increase of the backache, general malaise, and often menorrhagia. There is usually profuse leucorrhœa, if uncomplicated, glairy, not opaque.

It seems at first sight to be a trivial complaint, but, in proportion to the length of time it has existed, often proves very intractable, and necessitates a considerable amount of patience, both on the part of the medical attendant and the sufferer.

It is, however, a disease of considerable importance, as, if neglected, it becomes a source of much misery, and often a bar to pregnancy. Moreover, we are not in a position to disprove that, in patients inheriting a tendency to malignant disease, it is not a forerunner of epithelioma.

The majority of cases present themselves to us in the out-patient room, and, as hospital accommodation is necessarily limited, we cannot as a rule place out-patients in the most favorable conditions for rapid recovery. For the most part women suffering from granular inflammation are the poor, with large families, the consequent home duties rendering the needed rest almost an impossibility; and in our treatment we are therefore driven to attempt to cure with circumstances militating constantly against our utmost efforts. It is therefore scarcely to be wondered at if the results are so often unsatisfactory, and if we are tempted to pursue a course of treatment that may seem needlessly heroic, but which becomes imperative if we would successfully combat the disease under such unfavorable circumstances.

Endocervicitis is met with uncomplicated by granular inflammation of the lips of the uterus, though the granular condition exists in the cervical canal; but in cases of granular inflammation of the lips there is generally some endocervicitis.

In flexions of the uterus there is nearly always associated granular inflammation, that lip being affected which is on the side of the flexion; the posterior lip in cases of retroflexion, and the anterior in cases of antiflexion. This is doubtless due to the pressure on the side of the flexion producing hyperæmia of the lip on that side, which condition of hyperæmia being maintained tends to granular inflammation.

Granular inflammation is often a sign of the existence of submucous induration of the cervix, or areolar hyperplasia, though the latter disease may exist without it; but when this condition is present the granular inflammation only disappears when the subjacent induration is removed.

The treatment of granular inflammation of the cervix is tedious, especially when it has been allowed to run on for some months before advice is sought. To effect a cure it is imperative that the patient should rest entirely from exercise, and from sexual intercourse; she should if possible lie up for the greater part of the day, in the horizontal position, or if possible with the pelvis slightly elevated. The lips of the uterus should be freely scarified at intervals of about seven days, so as to lesson the local congestion, and in some cases it is necessary to carry the scarification to some considerable depth. After this has been repeated several times and ergot having been administered in doses of about *m* xx to *m* xxx of the liquid extract three times a day, some astringent caustic should be applied at intervals of from seven to ten days. Nitrate of silver, the common refuge in such cases, is of little use; in some cases doing positive harm. The liquor hydrargyri pernitratis should be used, taking due precaution that it does not run upon the vagina; and it will be found that with care a few applications of this will effect a cure. Fuming nitric acid may be substituted for this in some cases.

Strong carbolic acid (carbolic acid 9, water 1) is also of great use in promoting a healthy condition in the inflamed cervix. If, however, the granulations are unusually coarse, and do not yield to the above treatment, then the potass caustica lightly applied, so as to destroy the surface, will often induce the mucous membrane to put on a more healthy appearance. In some cases the application of the potassa is extremely painful. In these it is often a good plan to touch the affected part with the actual cautery; and since we have had introduced into England the excellent petroleum cautery of M. Paquelin, we possess a means of supplying the fer rouge with the least possible alarm to our patients, as the apparatus is contained in a small compass, and the preparations for its use do not involve the heating of irons at a fire.

It is much to be hoped that our increased knowledge of the pathology of this disease may lead to its more rational treatment, and that we may no longer hear of patients suffering from, and being under treatment for months for, so-called "ulcers of the womb."—*Obstretrical Journal.*

